# Food Value of Edible Fungi

**Published:** 1899-01

**Authors:** 


### FOOD VALUE OF EDIBLE FUNGI.

Mushrooms are chiefly of interest as a condiment, and their food value has been much exaggerated. A number of edible varieties have been analyzed, and their digestibility determined by methods of artificial digestion (*American Journal of Physiology*). The true digestible protein ranged from about 5 to 10 per cent.; watery element from 74 to 92 per cent. "In this respect they resemble ordinary vegetables, and the term 'vegetable-beefsteak,' which is often applied to them, is very erroneous. The carbohydrate content of the fungi is relatively high, but until more is known regarding the nature and digestibility of the carbohydrate constituents of various vegetable foods, it will be useless to draw comparisons. As dietetic accessories the edible fungi may play an important part, but investigation has demonstrated that they can not be ranked with the essential foods."—*Exchange.*

